# Novel Insights on the Corpus Luteum Function: Role of Vaspin on Porcine Luteal Cell Angiogenesis, Proliferation and Apoptosis by Activation of GRP78 Receptor and MAP3/1 Kinase Pathways

**DOI:** 10.3390/ijms21186823

**Published:** 2020-09-17

**Authors:** Patrycja Kurowska, Ewa Mlyczyńska, Joelle Dupont, Agnieszka Rak

**Affiliations:** 1 Department of Physiology and Toxicology of Reproduction, Institute of Zoology and Biomedical Research, Jagiellonian University in Krakow, 30-387 Krakow, Poland; patrycja.kurowska@doctoral.uj.edu.pl (P.K.); ewa.mlyczynska@alumni.uj.edu.pl (E.M.); 2 INRAE, UMR85, Unité Physiologie de la Reproduction et des Comportements, 37380 Nouzilly, France; joelle.dupont@inra.fr

**Keywords:** vaspin, ovary, corpus luteum, angiogenesis, apoptosis, proliferation

## Abstract

Formation and limited lifespan of corpus luteum (CL) are important for proper ovarian periodicity and fertility. Failed vascularization, imbalance between proliferation and apoptosis leads to luteal phase deficiency and infertility. The aim of this study was to examine the effect of vaspin on angiogenesis, apoptosis and proliferation as well as the involvement of 78-kDa glucose-regulated protein receptor (GRP78) and mitogen-activated kinase (MAP3/1) in these processes. Porcine luteal cells were incubated with vaspin (0.1–10 ng/mL) for 24 h to 72 h and then mRNA and protein expression of angiogenesis: vascular endothelial growth factor (VEGFA), fibroblast growth factor 2 (FGF2), angiopoietin 1 (ANGPT1), VEGFA receptors (VEGFR1, VEGFR2), apoptosis: caspase 3, bcl-2-like protein 4 (BAX), B-cell lymphoma (BCL2), and proliferation: proliferating cells nuclear antigen (PCNA), cyclin A factors as well as secretion of VEGFA, FGF2, ANGT1 were measured by real-time polymerase chain reaction (PCR), immunoblotting and enzyme-linked immunosorbent assay (ELISA), respectively. Moreover, apoptosis was assessed by caspase activity using the Caspase-Glo 3/7 assay, while proliferation was by alamarBlue. We found that vaspin enhanced luteal cell angiogenesis, proliferation, and significantly decreased apoptosis. Additionally, using GRP78 siRNA and the pharmacological inhibitor of MAP3/1 (PD98059), we observed that the effect of vaspin was reversed to the control level in all investigated processes. Taken together, our results suggest that vaspin is a new regulator of female fertility by direct regulation of CL formation and maintenance of luteal cell function.

## 1. Introduction

Formation and limited lifespan of corpus luteum (CL) is important for proper ovarian periodicity and consequently fertility [1]. In each reproductive cycle, the newly formed CL develops following ovulation, while CL from the previous cycle is eliminated in the process termed luteolysis [2]. Critical event for proliferation of CL and its establishment as an endocrine organ is vascular development [1]. Extensive capillary network efficiently supplies nutrients, hormones, and cholesterol to the luteal cells and provides output of progesterone (P4) [1]. Therefore, inappropriate vascularization leads to aberrant CL development and reduces the serum concentration of P4 [3], consequently causing miscarriages [4]. Angiogenesis is regulated by several factors including vascular endothelial growth factor (VEGFA), fibroblast growth factor 2 (FGF2), and angiopoietin 1 (ANGPT1). Both VEGFA and FGF2 promotes endothelial cell differentiation, proliferation, migration, and vascular tube formation [5], while ANGPT1 promotes the maturation and stabilization of nascent vessels [6]. Whereas in the absence of embryos, CL undergoes regression characterized by disrupt P4 production caused by increase in prostaglandin F (PGF2α) secretion, degeneration of both luteal and endothelial cells is observed [2]. Luteolysis is a feature of apoptosis including condensation and fragmentation of DNA and cell destruction mediated by caspase 3 as an answer to increased ratio of proapoptotic to antiapoptotic proteins on both mitochondrial and death receptors pathways [7]. Furthermore, imbalance between cell proliferation and apoptosis disturbs cellular homeostasis and leads to incorrect ovarian periodicity, cancerogenesis, and developmental abnormalities [8]. The aims of the research are to better understand the processes of CL establishment, formation, angiogenesis, and regression as well as molecular mechanisms in luteal cells function. Thus, the obtained data will contribute to solving the problem of infertility caused by CL failure.

Recent studies have increased our understanding of local factors associated with the development and regression of luteal cells. It is a well-known fact that vasoactive peptides, cytokines, and adipokines control luteal angiogenesis, apoptosis, or proliferation [9]. Belonging to vasoactive peptides, angiotensin II stimulated FGF2 mRNA levels in bovine luteal cells and increased the proliferation of endothelial cells [10]. Most common cytokines and tumor necrosis factor α were associated with both morphological and biochemical features of apoptosis in CL microvascular endothelial cells [11]. Additionally, Wiles et al. (2014) reported that the best described adipokine, leptin, upregulated the expression of VEGFA, FGF2, and ANGPT1 in goat cultured luteal cells [12], while in porcine, CL have antiapoptotic properties by decreasing caspase 3 activity [13].

Vaspin is an adipokine described in 2005 in the adipose tissue of Otsuka Long-Evans Tokushima Fatty (OLETF) rats [14]. Like most adipokines, vaspin action is linked with the regulation of energy balance like decreases in food intake [15], promotes preadipocyte differentiation [16], improves insulin sensitivity and glucose tolerance [14], and is postulated as a compensatory factor in type 2 diabetes [17]. A significant higher level of vaspin was observed in obese patients [18]. Connection between vaspin and reproduction was first mentioned, when Gonzalez et al. [19] described a higher level in serum of women than men, suggesting involvement of steroid hormones in the regulation of vaspin secretion. Our previously study described that expression of vaspin in the porcine adipose tissue and ovarian follicles was higher in fat Meishan pigs compared to lean Large White [20]. Moreover, both vaspin and its receptor, 78-kDa glucose-regulated protein (GRP78) expression, were increased in CL during the luteal phase [21]. Additionally, we noted that vaspin exerts a stimulatory effect on steroids P4 and estradiol (E2) secretion as well as the prostaglandin ratio of luteotropic PGE2 to luteolytic PGF2α in porcine luteal cells, suggesting its direct role in maintaining luteal secretory function [21]. Interestingly, positive association between serum vaspin level and angiogenesis intensity was observed in the human portal tracts and lobules in chronic hepatitis liver [22]. Additionally, the literature data show that in human endothelial cells, vaspin promotes proliferation and has antiapoptotic properties by activation of GRP78 and protein kinase B (AKT) [23]. Stimulatory effects of vaspin on granulosa cell proliferation, cell cycle progression, and inhibitory effect on apoptosis via mitogen activated kinase (MAP3/1), AKT, Janus kinase (STAT3), and GRP78 were noted by us [24]. Thus, in the present study, we hypothesize that vaspin is a luteotropic factor in the ovary, regulates CL formation as well as maintains luteal cell function by direct actions on luteal cell angiogenesis, proliferation, and apoptosis. Hence, the aims of the study were to investigate (i) the dose-dependent effect of vaspin on angiogenic factors VEGFA, FGF2, ANGPT1 mRNA expression and secretion as well as the VEGFR1 and VEGFR2 receptors protein level; (ii) the dose- and time-dependent effects of vaspin on apoptosis by evaluating caspase 3 and 7 enzyme activity as well as mRNA and protein expression of B-cell lymphoma (BCL2)/bcl-2-like protein 4 (BAX) and caspase 3; (iii) the dose- and time-dependent effect of vaspin on proliferation, proliferating cells nuclear antigen (PCNA), and cyclin A protein expression; and (iv) the involvement of the GRP78 receptor and kinase MAP3/1 on vaspin-mediated luteal cells’ angiogenesis, apoptosis, and proliferation.

## 2. Results

### 2.1. Dose-Dependent Effect of Vaspin on Luteal Cell Angiogenesis

**Figure 1:**
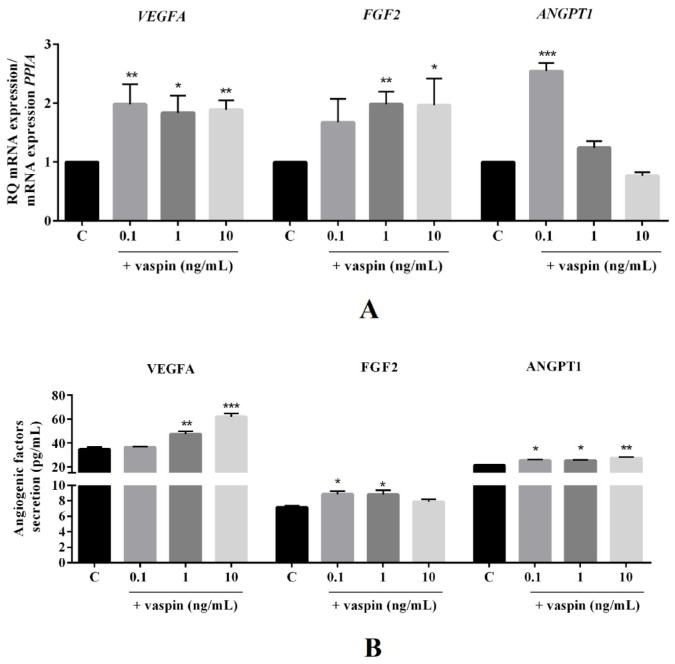

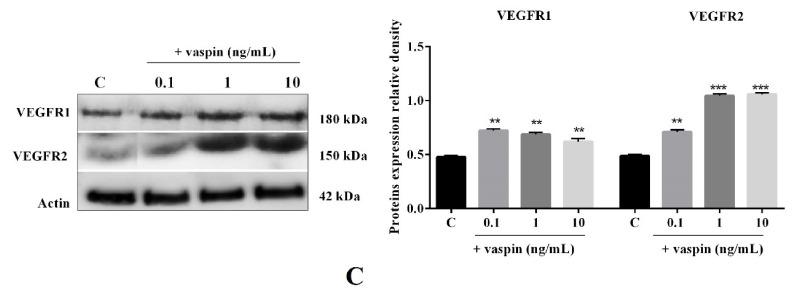
Dose-dependent effect of vaspin on luteal cell angiogenesis. The cells were treated with 0.1, 1, and 10 ng/mL of vaspin for 24 h, and then PCR, ELISA and western blot analysis were performed to determine mRNA levels and secretion of endothelial growth factor (VEGFA), fibroblast growth factor 2 (FGF2), and angiopoietin (ANGPT1) (**A**,**B**) or endothelial growth factor receptors 1 and 2 (VEGFR1 and VEGFR2) protein expression (**C**). Gene expression levels were normalized to cyclophylin A (PPIA), while proteins to actin. Experiments were independently performed and repeated four times (*n* = 4). The data are plotted as the means ± SEM. Significance between control and vaspin treatments is indicated by * *p <* 0.05, ** *p <* 0.01, and *** *p <* 0.001; Control (C).

Vaspin is linked with angiogenesis in human portal tracts and lobules in chronic hepatitis liver [22]. Vascularization is an essential process for proper CL formation [1]. Therefore, we investigated the dose-dependent (0.1, 1, and 10 ng/mL) effect of vaspin on luteal cells angiogenesis. We showed that vaspin significantly upregulated mRNA expression of VEGFA (2.0-, 1.8-, and 1.9-fold) at all investigated doses, FGF2 (2.0- and 1.9-fold) at 1 and 10 ng/mL, and ANGPT1 (2.5-fold) at 0.1 ng/mL compared to the control (* *p* < 0.05, ** *p* < 0.01, *** *p* < 0.001, Figure 1A). Similarly, enhancement in angiogenic factor secretion after vaspin treatment were observed at 1 and 10 ng/mL in VEGFA (47.51 ± 2.13 and 61.92 ± 2.75 vs. 34.77 ± 1.51 in control), at 0.1 and 1 ng/mL in FGF2 (8.86 ± 0.38 and 8.81 ± 0.53 vs. 7.1 ± 0.15 in control), and at all doses in ANGPT1 (25.37 ± 0.65, 25.27 ± 0.57, and 27.41 ± 0.81 vs. 21.34 ± 0.53 in the control) (* *p* < 0.05, ** *p* < 0.01, *** *p* < 0.001, Figure 1B). Additionally, we found a stimulatory effect of vaspin on the protein expression of VEGFR1 and VEGFR2 receptors at all examined doses (** *p* < 0.01, *** *p* < 0.001, Figure 1C), indicating vaspin as a new regulator of luteal cell angiogenesis.

### 2.2. Dose- and Time-Dependent Effect of Vaspin on Luteal Cell Apoptosis

**Figure 2:**
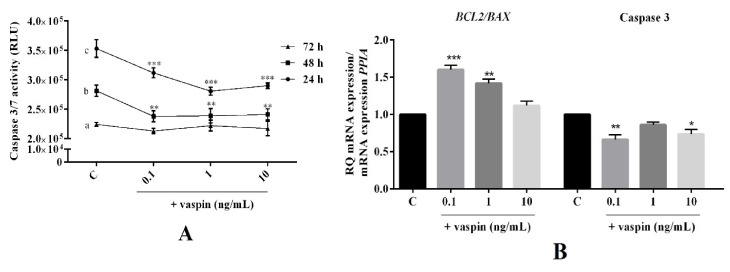

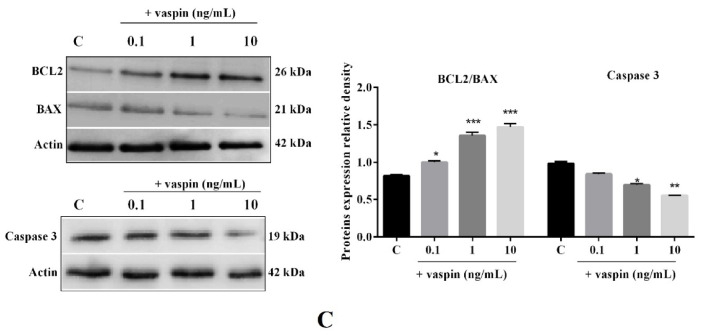
Dose- and time-dependent effect of vaspin on luteal cell apoptosis. The cells were treated with 0.1, 1, and 10 ng/mL of vaspin for 24 h to 72 h, after which caspase 3 and 7 activity (**A**) was analyzed using a Caspase-Glo 3/7 assay. Additionally, cells were incubated for 24 h with 0.1, 1, and 10 ng/mL of vaspin and then real-time PCR and western blot analysis were performed to determine BAX (bcl-2-like protein 4) BCL2 (B-cell lymphoma 2), and caspase 3 mRNA (**B**) and protein (**C**) expression. Genes expression levels were normalized to cyclophylin A (PPIA), while proteins to actin. Experiments were independently performed and repeated four times (*n* = 4). The data were plotted as the means ± SEM. Significance between the control and vaspin treatments is indicated by * *p* < 0.05, ** *p* < 0.01, and *** *p* < 0.01, while between different times of cultures by letters; Control (C), Relative Luminescence Units (RLU).

Vaspin is known for its antiapoptotic properties in porcine granulosa cells [24]. Hence, we examined the dose- (0.1, 1 and 10 ng/mL) and time- (24 h to 72 h) dependent effect of vaspin on luteal cell apoptosis. As shown in Figure 2, we observed that vaspin at all investigated doses significantly decreased caspase 3 and 7 activity after 24 h and 48 h of incubation, while had no effect after 72 h of incubation (** *p <* 0.01, *** *p <* 0.001, Figure 2A). Furthermore, after 24 h of incubation, vaspin at 0.1 and 1 ng/mL significantly increased the ratio of BCL2/BAX mRNA (1.6- and 1.4-fold), while at 0.1 and 10 significantly inhibited caspase 3 mRNA expression (1.7- and 1.4-fold) compared to the control (* *p <* 0.05, ** *p <* 0.01, *** *p <* 0.001, Figure 2B). Interestingly, a similar effect of vaspin was observed for protein level, upregulated ratio of BCL2/BAX at all investigated doses, while at 1 and 10 ng/mL, there was an inhibitory effect on cleavage caspase 3 protein expression (* *p <* 0.05, ** *p <* 0.01, *** *p <* 0.001, Figure 2C), suggesting that vaspin participates in luteal cell function maintenance.

### 2.3. Dose- and Time-Dependent Effect of Vaspin on Luteal Cell Proliferation

**Figure 3:**
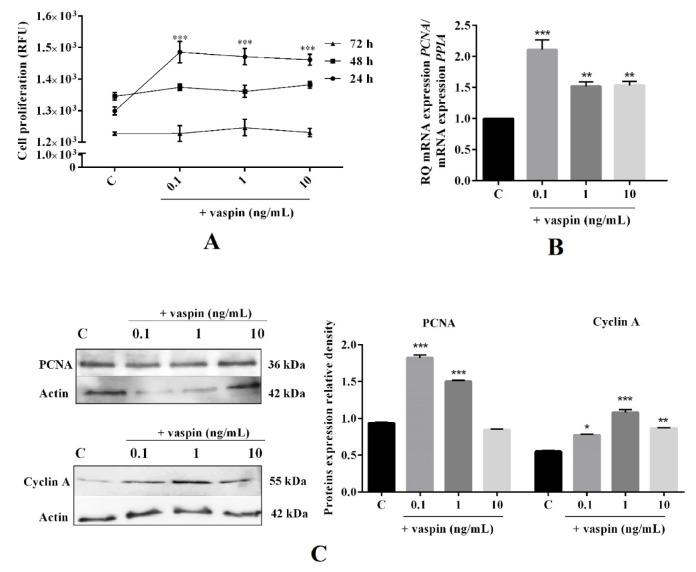
Dose- and time-dependent effect of vaspin on luteal cell proliferation. The cells were treated with 0.1, 1, and 10 ng/mL of vaspin for 24 h to 72 h, after which cell proliferation (**A**) was analyzed using the alamarBlue assay. Additionally, cells were incubated for 24 h with 0.1, 1, and 10 ng/mL of vaspin and then real-time PCR and western blot analysis were performed to determine PCNA (proliferating cells nuclear antigen) mRNA (**B**) and PCNA or cyclin A protein expression (**C**). Gene expression levels were normalized to cyclophylin A (PPIA), while proteins to actin. Experiments were independently performed and repeated four times (*n* = 4). The data were plotted as the means ± SEM. Significance between the control and vaspin treatments is indicated by * *p* < 0.05, ** *p* < 0.01, and *** *p* < 0.01; Control (C), Relative Fluorescence Units (RFU).

Vaspin was also described as a positive regulator of proliferation in the granulosa cells [24]. Thus, we determined the dose- (0.1, 1 and 10 ng/mL) and time- (24 h to 72 h) dependent effects of vaspin on luteal cell proliferation. We demonstrated, that vaspin at all studied doses significantly increased luteal cell proliferation after 24 h of culture, however, had no effect after 48 h and 72 h (*** *p* < 0.01, Figure 3A). Moreover, after 24 h of incubation, vaspin at all investigated doses significantly increased PCNA mRNA expression (2.1-, 1.5, and 1.5-fold) compared to the control (** *p* < 0.01, *** *p* < 0.001, Figure 3B). Interestingly, a similar effect of vaspin was observed for the PCNA protein level and vaspin at all investigated doses stimulated cyclin A protein expression (* *p* < 0.05, ** *p* < 0.01, *** *p* < 0.001, Figure 3C), confirming the direct effect of vaspin on luteal proliferation.

### 2.4. Involvement of the GRP78 Receptor and MAP3/1 Kinase in the Effects of Vaspin on Luteal Cells

**Figure 4:**
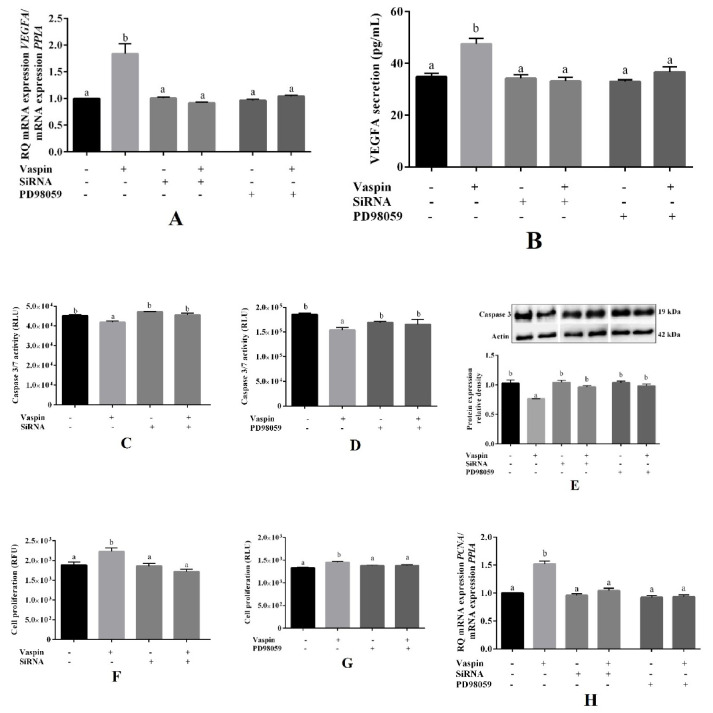
Involvement of the 78-kDa glucose-regulated protein (GRP78) receptor and mitogen activated kinase (MAP3/1) on the effect of vaspin on luteal cells. Cells were pretreated with GRP78 siRNA (2 nM) for 24 h or with PD98059 (5 μM) MAP3/1 kinase inhibitor for 1 h; afterward, vaspin (1ng/mL) was added for 24 h, then VEGFA (vascular endothelial growth factor) (**A**), VEGFA secretion (**B**), caspase 3 and 7 activity (**C**,**D**), caspase 3 protein level (**E**), cell proliferation (**F**,**G**), PCNA (proliferating cells nuclear antigen) mRNA expression (**H**) were determined by using real-time PCR, ELISA, Caspase-Glo 3/7 assay, western blot and alamarBlue assay. Gene expression levels were normalized to cyclophylin A (PPIA), while protein to actin. Experiments were independently performed and repeated four times (*n* = 4). The data were plotted as the means ± SEM. Significance between control and vaspin or inhibitors and vaspin + inhibitors treatments are indicated by different letters (*p* < 0.05); Control (C), Relative Fluorescence Units (RFU), Relative Luminescence Units (RLU), + with reagent, - without reagent.

Previous data have shown that vaspin regulated GRP78 expression and significantly stimulated phosphorylation of MAP3/1 kinase in porcine luteal cells [21]. Therefore, we examined the role of GRP78 receptor and MAP3/1 kinase in vaspin-mediated effects on luteal cell angiogenesis, apoptosis, and proliferation. VEGFA mRNA expression and VEGFA secretion, caspase 3/7 activity, and cleavage caspase 3 protein level, cell proliferation, and PCNA transcript were assessed after 24 h of incubation with GRP78 siRNA (2 nM) or a pharmacological inhibitor of MAP3/1 kinase PD98059 (5 μM). We indicated that simultaneous treatment with GRP78 siRNA or PD98059 added with vaspin (1 ng/mL) reversed all investigated factors to the control level (*p* < 0.05, Figure 4). Furthermore, we noted that GRP78 siRNA and PD98059 added alone had no effect on the studied processes. These results clearly document the molecular mechanism of vaspin action on angiogenesis, apoptosis, and proliferation in the luteal cells.

## 3. Discussion

Formation of CL and the establishment of luteal cell secretory function are key, closely regulated processes in mammalian reproduction essential for proper fertility and maintaining pregnancy [25]. Our previous findings indicated vaspin as luteotropic factor in luteal cells by upregulating P4 production as well as PGE2 secretion [21]. In the present study, we confirmed the mentioned hypothesis showing the stimulatory effects of vaspin on luteal angiogenesis. The development of CL is accompanied by rapid angiogenesis; new vessels are formed from preexisting ones. The capillary network efficiently supplies nutrients, hormones, and cholesterol to the luteal cells and endocrine function develops quickly with an efficient output of P4 [1]. More precisely, our data showed that vaspin enhanced angiogenic factors like VEGFA, FGF2, and ANGPT1 mRNA expression and secretion by porcine luteal cells during 24 h of culture. We hypothesize that the stimulatory effect of vaspin on luteal angiogenesis is through the direct action of vaspin on mRNA expression of angiogenic factors, independent of the properties of mitogenic vaspin. Indeed, in the present studies, we observed differences at the mRNA level of angiogenic factors and its protein levels, suggesting translational regulations of VEGFA, FGF2, and ANGPT1. Genome-wide poor correlation between expression levels of mRNA and protein are commonly reported and may not be mutually exclusive [26,27]. There are many complicated and varied post-transcriptional mechanisms involved in turning mRNA into protein that are not yet sufficiently well-defined to be able to compute protein concentrations from mRNA. It is a well-known fact that VEGFA is the most remarkable regulator of angiogenesis in the CL [28] and promotes proliferation, survival, and chemotaxis of endothelial cells isolated from steroidogenic tissues [29]. ANGPT1 stimulates sprouting and maturation of blood vessels [29]. Interestingly, FGF2 antibodies suppress endothelial cells’ proliferative activity, P4 production, and luteal volume in pigs [5,30], suggesting its importance for CL formation and secretory function. In our study, we showed stimulatory effects of vaspin on the protein expression of VEGFR1 and VEGFR2 receptors, which have a necessary function in CL formation, for example, in bovine, CL inhibition of VEGFR2 leads to a reduction of 52% in the endothelial network [31]. In our experiments, we performed luteal cell culture collected from CL at the middle luteal phase—the most stable phase of mature CL-with full secretory capacity [32]. Our findings are important considering that inappropriate vascularization is associated with inadequate luteal function, reducing the circulating concentration of P4, and leading to infertility and early embryonic loss [1]. Furthermore, the results are with good agreement with data indicating a positive association between serum vaspin level and angiogenesis intensity in human portal tracts and lobules [22]. Correlation between adipose tissue, indirect obesity, and vascularization has been found before for leptin, which is known for in vitro stimulation of angiogenesis in goat luteal cells [12].

Concerning the above, in the next experiments, we focused on better understanding the vaspin effect on CL development. The results of our studies showed that vaspin decreased luteal cell apoptosis and stimulated proliferation. Balance between apoptosis and proliferation is essential for proper fertility; luteal cell apoptosis is a key component in the process of structural CL regression, which ultimately leads to luteal cell death and new cycle may begin [33], whereas proliferation is essential for luteal cell growth and blood vessel formation [2]. Abnormalities in apoptosis and proliferation lead to ovarian pathologies like infertility, generation of cancer and luteal phase deficiency, finally causing miscarriages [34]. Our data showed that vaspin decreased caspase 3 and 7 activity after 24 h and 48 h and protein levels at 24 h of incubation as well as increased the ratio of antiapoptotic BCL2 to proapoptotic BAX expression. BAX acts to permeabilize the mitochondria, allowing the release of cytochrome c, activate caspase 9. and then the executioner caspase 3 [35]. Activation of caspase 3 is a central event in the apoptotic process upon which numerous signaling pathways converge and through which multiple downstream substances are cleaved [35]. Furthermore, the ratio of BAX/BCL2 and expression of caspase 3 in luteal cells increases in the late luteal phase compared to the early luteal phase [33], indicating its participation in the structural luteolysis of porcine CL [7]. Our results clearly document that vaspin, by direct effect on luteal apoptosis, can be a novel regulatory factor on CL regression and can prevent luteal phase deficiency. Additionally, to confirm our hypothesis, we noted that vaspin directly stimulated PCNA and cyclin A level. The PCNA protein is a proliferation marker; the highest accumulation was observed in the S proliferator phase of cell cycle [36] and during CL formation compared to atretic CL [37]. Furthermore, proliferation is closely related to cell cycle progression, which is regulated by cyclins [38]. Cyclin A is a well-established marker of porcine cell proliferation, growth, and development, and which level research as a peak in the S phase of the cell cycle [39]. Our results is in a good agreement with data obtained in porcine granulosa, where vaspin decreased apoptosis and caspase 3 level, while increased BAX/BCL2, cyclin A expression, and proliferation [24]. Interestingly, our results can also be partly explained by our previously study where vaspin stimulated luteal P4 secretion, which has antiapoptotic properties [40] and significantly decreased PGF2α levels, whose upregulation led to luteal cell apoptosis [33]. In the present study, we observed differences between mRNA and proteins of the proliferator or apoptotic factor expression like BAX. The literature data showed similar inconsistencies, for example, Przygrodzka et al. [7] described an increase in tumor necrosis factor α mRNA expression with no effect on protein, while Rak et al. [41] found a stimulatory effect of resistin on BCL2 protein with no effect on gene expression, suggesting differences in post-transcriptional regulation or protein stability in ovarian cells [41]. Additionally, we noted that the effect of vaspin on cell proliferation and apoptosis were dependent on the time of cell incubation. We observed that vaspin decreased apoptosis after 24 h and 48 h of incubation, while increased cell proliferation after 24 h, which is in good agreement with our previous studies where vaspin affected cell apoptosis and proliferation during 24 h and 48 h of incubation in granulosa cells [24]. Moreover, several literature data have documented the quick (from 12 to 48 h) action/activation of vaspin on cell proliferation or apoptosis (e.g., 24 h in cardiomyocytes [42], 12 h in mesenchymal stem cells [43], or 48 h in human osteoblasts [44]). To summarize, the obtained results indicated that vaspin, like leptin [13], regulates porcine luteal apoptosis and proliferation and establishes CL development. 

Finally, we examined the molecular mechanism of vaspin action in luteal angiogenesis, apoptosis, and proliferation. Using GRP78 siRNA and a pharmacological inhibitor of MAP3/1, we demonstrated that the stimulatory effect of vaspin on angiogenesis (VEGFA mRNA expression and VEGFA secretion) or proliferation (PCNA mRNA expression) and inhibitory action on apoptosis (caspases activity and protein expression) was reversed to the control level. GRP78 is present on the cell surface where it acts as a receptor and regulates cellular proliferation and survival and is also involved in reproductive processes including follicular, CL, embryo, and preimplantation development [45]. In human umbilical vein endothelial cells, after GRP78 silencing, the angiogenesis-related pathway proteins VEGF/VEGFR were reduced [46]. Moreover, our previously studies showed that vaspin regulated endocrinology, especially steroid secretion from both ovarian follicles and luteal cells by activation of the GRP78 receptor [21,47]. The mitogenic and prosurvival activities of VEGFA correlated with the ability of this peptide to induce phosphorylation of MAP3/1 in adrenal cortex capillary endothelial cells [29]. Moreover, the MAP3/1 pathway promotes cell death by the activation of death receptors, cytochrome c release, or upregulation of BAX [48]. Our results are in good agreement with data describing that vaspin stimulated granulosa cell proliferation and decreased apoptosis by activation of the GRP78 receptor and MAP3/1 kinase pathways [24]. Moreover, in mouse mesenchymal stem cells [43] and in human osteoblasts [44], vaspin protects from apoptosis through the MAPK/p38 pathway. Furthermore, activation of MAP3/1 was also described in prosurvival action of resistin [41] and proliferator ability of apelin [49] in porcine ovarian follicles.

## 4. Materials and Methods

### 4.1. Reagents

Phosphate-buffered saline (PBS) (product no. 14040174), fetal bovine serum (FBS) (product no. 16140071), Lipofectamine 3000 (product no. L3000001), GRP78 siRNA, the TaqMan Gene Expression Cells-to-CT Kit (product no. AM1728), and electrophoresis markers were obtained from ThermoFisher Scientific (Waltham, MA, USA). Medium M199 (product no. M2154), antibiotic-antimycotic solution (product no. A5955), trypsin (product no. T4049), vaspin (product no. SRP4915), Laemmli buffer (product no. 38733), Tris, sodium dodecyl sulfate (SDS), and Tween 20 were purchased from Sigma-Aldrich (St. Louis, MO, USA). PD98059 (product no. 1213) was obtained from Tocris (Bristol, UK GB). 4–20% gels (product no. 456-1093) and membranes (product no. 1704156) were bought from Bio-Rad (Hercules, CA, USA). WesternBright Quantum HRP substrate (product no. K-12043 D200) was purchased from Advansta Inc. (Menlo Park, CA, USA).

### 4.2. Luteal Cells In Vitro Culture

Porcine ovaries were collected from Polish Large White sexually mature animals (6–8 months old) at a local abattoir under veterinarian control and then transported to the laboratory in PBS with antibiotic-antimycotic solution within 30 min of collection. Therefore, an agreement of ethical commission was not necessary. Sows were euthanized during slaughter according to European Legislation (EFSA, AHAW/04-027). Based on results from vaspin/GPR78 expression (Kurowska et al. 2020) we collected CL from the middle stage of the estrous cycle. Luteal cell cultures were prepared according to Gregoraszczuk [50], as also provided previously [51]. Briefly, after isolation by trypsinization, cells were resuspended in M199 supplemented with 10% FBS [*v*/*v*] and viability of the cells was determined using Trypan blue dye (95% of viable cells). Subsequently, cells were seeded in 96-well tissue culture plates at a concentration of 6 × 10^4^ viable cells/well for 24 h. All cultures were maintained at 37 °C in a humidified atmosphere consisting of 5% CO_2_/95% O_2_. 

#### Experimental Procedures

In the first experiment, luteal cells were incubated for 24 h in M199 supplemented with 1% FBS [*v*/*v*] as a control medium or containing vaspin at a concentration of 0.1, 1, and 10 ng/mL. Doses of vaspin were chosen based on our previous papers [21,24]. After incubation, the culture medium was stored at −20 °C for the quantification of VEGFA, FGF2, and ANGPT1 secretion. Luteal cells were washed in PBS and stored at −70 °C for analysis of VEGFA, FGF2, ANGPT1, caspase 3, BAX, BCL2, and PCNA mRNA expression or boiled in Laemmli buffer for 4 min for the measurement of VEGFR1 and VEGFR2, caspase 3, BAX, BCL2, PCNA, and cyclin a protein expression.

To determine time- and dose-dependent effects of vaspin on cell proliferation and caspases 3 and 7 activity, luteal cells were incubated for 24, 48, or 72 h in M199 medium supplemented with 1% FBS [*v*/*v*] and vaspin at doses 0.1, 1, 10 ng/mL. Subsequently, alamarBlue reagent was added for 2 h to evaluate cell proliferation, while the Caspase-Glo 3/7 assay for 1.5 h was used to assess the caspases 3 and 7 activity. 

**Figure 5:**
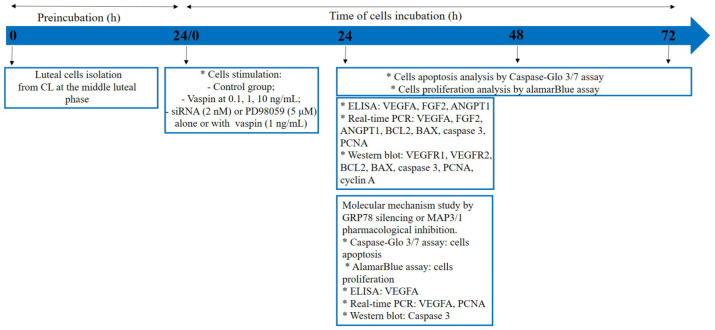
Experimental design graph; CL (corpus luteum), BCL2 (B-cell lymphoma), BAX (bcl-2-like protein 4), PCNA (proliferating cells nuclear antigen), VEGFA (vascular endothelial growth factor), FGF2 (fibroblast growth factor 2), ANGPT1 (angiopoietin 1), GRP78 (78-kDa glucose-regulated protein) MAP3/1 (mitogen activated kinase), and VEGFR1/2 (endothelial growth factor receptors), * scoring/pause.

To investigate the molecular mechanism of vaspin action on luteal cell function, cells were incubated in M199 without FBS for 24 h and next pre-treated with GRP78 siRNA at 2 nM for 24 h. To investigate the involvement of MAP3/1 kinase, luteal cells cultured in M199 supplemented with 1% [*v*/*v*] FBS were pre-treated for 1 h with a pharmacological inhibitor of MAP3/1, PD98059, at a dose of 5 μM [21]. Subsequently, vaspin at a concentration of 1 ng/mL was added for the next 24 h and then the medium was stored at −20 °C for VEGFA secretion, while cells were washed in PBS and stored at −70 °C for VEGFA and PCNA expression or boiled in Laemmli buffer for 4 min for caspase 3 protein expression analysis. Additionally, to study cell proliferation, alamarBlue was added for 2 h or to evaluate cell apoptosis, Caspase-Glo 3/7 reagent was added for 1.5 h (Figure 5). 

### 4.3. Gene Silencing

GRP78 silencing in porcine luteal cells was designed according to the rules described by Park et al. [52] and based on our previous paper [21]. Luteal cells were transfected with GRP78 siRNA (2 nM) using Lipofectamine 3000 according to the manufacturer’s instructions and a neutral effect of Lipofectamine 3000 on porcine ovarian cells was checked prior to the experiments [21,47]. The sequences of siRNA against GRP78 used here were #909: CCU UCU CAC CAU UGA UAA UTT (sense), AUU AUC AAU GGU GAG AAG GTT (antisense); #693: GGG AAA GAA GGU UAC UCA UTT (sense), AUG AGU AAC CUU CUU UCC CTT (antisense); and #1570: GCC UCU GAU AAU CAG CCA ATT (sense), UUG GCU GAU UAU CAG AGG CTT (antisense).

### 4.4. AlamarBlue Assay

The alamarBlue assay (product no. DAL1100, Invitrogen, Carlsbad, CA, USA) was used to determine luteal cell proliferation based on the quantitation of cell metabolic activity, as described previously [24]. Briefly, blue, non-fluorescent resazurin was metabolically conversed to a pink, fluorescent resorufin by living cells. AlamarBlue stock solution was aseptically added to wells in amounts equal to 10% [*v*/*v*] of the incubation volume. After 2 h incubation with alamarBlue, the fluorescence was determined by measuring absorbance at 570 and 600 nm wavelengths using a FLUORO reader (BioTek Instruments, Winooski, VT, USA).

### 4.5. Caspase-Glo 3/7 Assay

The Caspase-Glo 3/7 assay (product no. G8090, Promega, Madison, WI, USA) was used to check caspases 3 and 7 activity. Briefly, the addition of Caspase-Glo 3/7 assay resulted in cell lysis, followed by caspase cleavage of the substrate. Substrate released aminoluciferin, which was consumed by luciferase and then a “glowing” luminescent signal proportional to caspases 3 and 7 activity was generated. The Caspase-Glo 3/7 assay stock solution was aseptically added to wells in amounts equal to 100% [*v*/*v*] of the incubation volume as previously described [24]. After 1.5 h of incubation, luminescence was measured at a 495 nm wavelength using a luminometer (SpectraMax L 147) and SoftMax Pro software (software version 7, Molecular Devices, San Jose, CA, USA).

### 4.6. Real-Time PCR

A TaqMan Gene Expression Cells-to-CT Kit was used for RNA isolation and cDNA synthesis following the manufacturer’s protocols. RNA and cDNA quantity were evaluated by measuring absorbance at the 260 nm and 280 nm wavelengths by spectrophotometry. TaqMan specific primers and TaqMan Gene Expression Master Mix were used, then amplifications were performed using the StepOnePlus system (Applied Biosystems, Carlsbad, CA, USA) following the manufacturer’s instructions and our previous paper (Kurowska et al. 2019). Studied genes (ThermoFisher Scientific, Waltham, MA, USA) were: *VEGFA* (product no. Ss03393993_m1), *FGF2* (product no. Ss03375809_u1), *ANGPT1* (product no. Ss03391075_m1) caspase 3 (product no. Ss03382792), *BAX* (product no. Ss03375842) and *BCL2* (product no. Ss03375167), and *PCNA* (product no. Ss03377029_g1). Cyclophylin A (*PPIA*) (product no. Ss03394782_g1) was used as the housekeeping gene. Briefly, quantitative PCR was performed with 100 ng cDNA, 1 TaqMan GeneExpression primers, and 10 TaqMan PCR master mix (Applied Biosystems) in a final reaction volume of 20. After a 2 min incubation at 50 °C, thermal cycling conditions were 10 min at 95 °C, followed by 40 cycles of 15 s at 95 °C and 1 min at 60 °C to determine the cycle threshold number (Ct) for quantitative measurement. The relative mRNA expression levels of apoptosis genes relative to *PPIA* were determined using the 2^−ΔΔCq^ method [53].

### 4.7. Western Blot

Western blotting, migration, and transfer were performed as previously described [24,51]. For each sample, 30–50 μg of protein was used for western blot. Primary and secondary antibodies were described in Table 1. Actin was used as a loading control after membrane stripping. Chemiluminescence signals were detected by the WesternBright Quantum HRP substrate and visualized using the Chemidoc XRS + System (BioRad, Hercules, CA, USA). Densitometry and ImageJ software (US National Institutes of Health, Bethesda, MD, USA) were used to quantify all visible bands.

### 4.8. Enzyme-Linked Immunosorbent Assay (ELISA)

Porcine VEGFA (product no. ab218298, Abcam, Cambridge, GB), FGF2 (product no. E07F0003, BlueGene Biotechnology, Shanghai, China), and ANGPT1 (product no. EP0202, FineTest, Wuhan, China) ELISA kits were used to measure vaspin effect on angiogenic factor secretion. The range of the kits were 0–1000 pg/mL for VEGFA and FGF2, while 0–5000 pg/mL for ANGPT1. The inter- and intra-experimental coefficients of variation were respectively <12% and <10% for VEGFA, <10% and <8% for FGF2, and <6.5% and <4.9% for ANGPT1. All samples were assayed in triplicate. Absorbance was measured at the 450 nm wavelength using an ELx808 ELISA microplate reader (BioTek Instruments, Winooski, VT, USA).

### 4.9. Statistical Analysis

Statistical data were presented as the means ± standard error of the mean (SEM) of four independent experiments. For one culture, luteal cells were collected from six ovaries (one ovary from pig), so the total number of animals was 72. Distribution of normality was checked by the Shapiro–Wilk test. One-way ANOVA was used for multiple competitions involving more than two treatment groups, and the Tukey test was used post-hoc (PRISM software version 5; GraphPad, La Jolla, CA, USA). Statistical significance is indicated by different letters (*p <* 0.05) or * *p <* 0.05, ** *p <* 0.1, *** *p <* 0.01.

## 5. Conclusions

**Figure 6:**
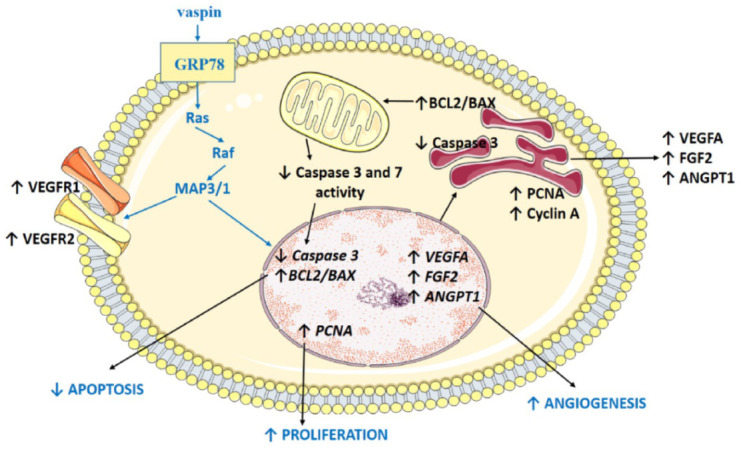
Model of vaspin regulatory action in porcine luteal cells. Vaspin stimulates angiogenesis and proliferation, while it inhibits apoptosis by the activation of 78-kDa glucose-regulated protein (GRP78) receptors and mitogen activated kinase (MAP3/1); BCL2 (B-cell lymphoma), BAX (bcl-2-like protein 4), PCNA (proliferating cells nuclear antigen), VEGFA (vascular endothelial growth factor), FGF2 (fibroblast growth factor 2), ANGPT1 (angiopoietin 1), ↑ stimulation, ↓ decreasing.

To conclude, our results showed that vaspin had a positive effect on angiogenesis and proliferation, while it decreased apoptosis in luteal cells by activation of the GRP78 receptor and MAP3/1 kinase pathways (Figure 6). This study indicates that vaspin is a new regulator in female fertility by the direct action on CL formation and luteal cell function maintenance. These data bring new knowledge on the regulation of ovarian physiology and could potentially lead to therapeutic interventions for infertility caused by inappropriate CL development, luteal phase deficiency, or irregular periodicity.

## Figures and Tables

**Table 1:** Antibodies used in western blot reaction. VEGFR1/2 vascular endothelial growth factor receptors, BCL2 (B-cell lymphoma), BAX (bcl-2-like protein 4), PCNA (proliferating cells nuclear antigen).

Antibody	Host	Dilution	Vendor
VEGFR1	rabbit	1:250	Abcam, GB, product no. ab2350
VEGFR2	rabbit	1:250	Abcam, GB, product no. ab45010
caspase 3	rabbit	1:1000	Cell Signaling Technology, USA, product no. 9662S
BCL2	rabbit	1:1000	Cell Signaling Technology, USA, product no. 4223S
BAX	rabbit	1:1000	Cell Signaling Technology, USA, product no. 2772S
cyclin A	mouse	1:1000	Cell Signaling Technology, USA, product no. 4656S
PCNA	mouse	1:250	ThermoFisher Scientific, USA, product no. 13-3900
actin	mouse	1:5000	Sigma-Aldrich, USA, product no. A5316
anti-rabbit	goat	1:1000	Cell Signaling Technology, USA, product no. 7074
anti-mouse	horse	1:1000	Cell Signaling Technology, USA, product no. 7076

## Abbreviations

AKT	protein kinase B
ANGPT1	angiopoietin 1
BAX	bcl-2-like protein 4
BCL2	B-cell lymphoma
C	control
CL	corpus luteum
E2	estradiol
FBS	fetal bovine serum
FGF2	fibroblast growth factor 2
GRP78	78-kDa glucose-regulated protein
MAP3/1	mitogen activated kinase
OLETF	Otsuka Long-Evans Tokushima Fatty
P4	progesterone
PBS	phosphate-buffered saline
PCNA	proliferating cells nuclear antigen
PGE2	prostaglandin E2
PGF2α	prostaglandin F2 alpha
PPIA	cyclophylin A
RFU	relative fluorescence units
RLU	relative luminescence units
SDS	sodium dodecyl sulfate
SEM	standard error of mean
STAT3	Janus kinase
VEGFA	vascular endothelial growth factor
VEGFR1/2	endothelial growth factor receptors
